# The relationship between parental mental health, reflective functioning coparenting and social emotional development in 0-3 year old children

**DOI:** 10.3389/fpsyg.2023.1054723

**Published:** 2023-05-30

**Authors:** Mia De Palma, Rosanna Rooney, Elizabeth Izett, Vincent Mancini, Robert Kane

**Affiliations:** ^1^Discipline of Psychology, School of Population Health, Curtin University, Perth, WA, Australia; ^2^Telethon Kids Institute, Perth, WA, Australia; ^3^Division of Paediatrics, UWA Medical School, University of Western Australia, Perth, WA, Australia

**Keywords:** parental reflective functioning, coparenting, child social emotional development, reflective functioning, parental mental health, child development and infant mental health

## Abstract

**Introduction:**

The transition to parenthood is a high-risk period for many parents and is an important period for child development. Research has identified that parental mental health, reflective functioning (capacity to consider mental states of oneself and others) and coparenting (capacity to work together well as a parenting team) may be particularly significant predictors of later child outcomes, however these factors have seldom been considered together. The present study therefore aimed to investigate the relationship between these factors and the extent to which they predict child social emotional development.

**Methods:**

Three hundred and fifty parents of infants aged 0 to 3 years 11 months were recruited to complete an online Qualtrics questionnaire.

**Results:**

Results indicate that both positive coparenting and parental reflective functioning (Pre-mentalizing and Certainty subscales) were found to significantly predict child development. General reflective functioning (Uncertainty subscale) predicted parental depression and anxiety, however unexpectedly, parental mental health was not a significant predictor of child development, but did predict coparenting. General reflective functioning (Certainty subscale) was also found to predict coparenting, which in turn was found to predict parental reflective functioning. We found an indirect effect of general reflective functioning (Certainty) on child SE development via parental reflective functioning (Pre-mentalizing). We also found an indirect effect of negative coparenting on child development via parental reflective functioning (Pre-mentalizing).

**Discussion:**

The current results support a growing body of research highlighting the important role reflective functioning plays in child development and wellbeing as well as parental mental health and the interparental relationship.

## Introduction

It is widely understood that the first 1,000 days of life—the period of development from conception to age two—is one of the most crucial periods of development for a child (Moore et al., 2017). Given the importance of this developmental period, it is thought that adverse experiences during this time may be particularly harmful for the child’s ongoing social emotional development, with consequences potentially spanning the child’s lifetime (Lyons-Ruth et al., 2017; Moore et al., 2017). Social emotional development in infancy entails the gradual increase in emotion recognition and expression, and participation in social interaction (Halle and Darling-Churchill, 2016). It is important to consider which factors in particular place a child at increased risk of adverse outcomes (Newland, 2015).

The transition to parenthood is accompanied by a series of novel and pre-existing stressors, and an increased demand on psychosocial resources that brings with it a greater risk of developing mental health difficulties for parents (Nyström and Öhrling, 2004). Within Australia, 21% of adults meet the diagnostic criteria for a mental health disorder (Australian Bureau of Statistics, 2020-21), and both maternal and paternal depression and anxiety are linked with a number of adverse child outcomes (McCall-Hosenfeld et al., 2016). These include delayed social (Ip et al., 2018), emotional (Kingston and Tough, 2014), behavioral and cognitive development (Kingston et al., 2012), lower ability to self-regulate (Hernández-Martínez et al., 2008), a more difficult temperament (Werner et al., 2007; Parfitt et al., 2013) and developmental delays (Davis and Sandman, 2010). Research has also found an increase in rates of internalizing and externalizing symptoms (Kane and Garber, 2004; Verbeek et al., 2012; Matijasevich et al., 2015) as well as depression (Murray et al., 2011; Pearson et al., 2013), among children of parents with perinatal depression or anxiety. Poor parental mental health has further been linked with difficulties in the parent-infant relationship (Murray et al., 2011; Lilja et al., 2012; Verbeek et al., 2012; Pearson et al., 2013; Matijasevich et al., 2015).

The importance of the parent infant relationship has been particularly emphasized within the field of attachment, with research consistently finding links between secure parent-infant attachment relationships and child outcomes such as positive mental health, social and emotional intelligence, physical health and enhanced cognitive capacity later in life (Ranson and Urichuk, 2008; Boldt et al., 2014). Caregiver sensitivity/responsiveness to an infant’s needs has been associated with attachment security, as has the parent’s own attachment representations (van Ijzendoorn, 1995; O'Neill et al., 2021). Parental attachment has been also linked with parenting behaviors, whereby secure attachment correlated with more positive parenting behaviors (Huang, 2021). Furthermore, attachment relationships are known to pass from parent to child, across generations (Steele and Steele, 2008; Sette et al., 2015).

Parental Reflective functioning (PRF) is a proposed mechanism through which these attachment relationships are transmitted from parent to child (Kelly et al., 2005; Steele and Steele, 2008). Reflective functioning, also referred to as mentalization, is defined as one’s ability to understand and link mental states with behavior both for oneself and for others (Slade, 2005; Stacks et al., 2014). Psychodynamic theorists assert that reflective functioning is involved in the development and maintenance of psychopathology including depression (Luyten et al., 2013; Luyten and Fonagy, 2018) and anxiety (Lavoie et al., 2014). They suggest that while in a depressed state, individuals may be significantly biased in their reflective processes and are typically not able to engage in reflective thinking (Luyten et al., 2013; Luyten and Fonagy, 2018). A lack of reflective capacity is also thought to prevent an individual from regulating their intense emotional experiences or modulating the behavioral expression of these emotions (Bouchard et al., 2008). These suggestions are supported by a body of research finding associations between poorer reflective functioning and higher depression levels across varying samples (Fischer-Kern et al., 2013; Belvederi Murri et al., 2017; Bigelow et al., 2018; Wendelboe et al., 2021). Interventions targeting reflective functioning have also been found to have a small effect in reducing both general and interpersonal distress symptoms (Hayden et al., 2018).

High levels of PRF is thought to be essential to children’s ability to regulate their emotions, and develop secure attachment relationships (Ordway et al., 2015). In fact, reflective functioning has been found to mediate the relationship between parental attachment and child social emotional wellbeing (Nijssens et al., 2020). Research suggests that parental reflective functioning allows parents to more consistently and sensitively respond to cues from their infant (Stacks et al., 2014). Moreover, poor maternal reflective functioning has been linked with adverse child outcomes including anxiety, externalizing behaviors, poor social competence and difficulty regulating emotions (Camoirano, 2017; Colonnesi et al., 2019). Other recent studies have shown that both maternal and paternal reflective functioning are linked with better social emotional adjustment (Gordo et al., 2020; Salo et al., 2021), enhanced social competence and higher levels of reflective functioning among adolescents (aged 14–18 years; Benbassat and Priel, 2012). Lower levels of reflective functioning in both parents have also been linked with more dysfunctional parent–child interactions (Vismara et al., 2021).

More recent research seeks to move beyond maternal–infant relationships to consider how the broader family system impacts a developing child. Family systems theory suggests that family-level processes influence child wellbeing over and above dyadic relationships within the family (i.e., the couple relationship, parent–child relationship and sibling relationships; Boričević Maršanić and Kušmić, 2013). Coparenting is a concept nested within family systems theory, and focuses on the intersection between parents’ romantic relationship and their new role as a parent (Salo et al., 2021). Correlational research has found that coparenting predicts unique variance in child social emotional development, and argues coparenting may have a larger impact on social emotional development than that of general parenting and the couple relationship alone (Feinberg and Kan, 2008; Boričević Maršanić and Kušmić, 2013). Coparenting is defined as a parents’ ability to work in harmony as a team for their child’s benefit (Le et al., 2016). When coparenting works, parents are able to come together and agree on how to parent their child, making coparenting a key predictor of overall family functioning (Dollberg et al., 2021). Feinberg (2003) describes a model of coparenting comprised of 7 dimensions, 5 encapsulating positive coparenting (coparenting agreement, coparenting closeness, coparenting support, endorsement of partner parenting, division of labor) and two which make up negative coparenting (exposure to conflict, and coparenting undermining).

Positive coparenting is associated with a variety of child outcomes including cognitive development (Shai, 2019) psychological and social emotional wellbeing (Teubert and Pinquart, 2010), social skills (Cabrera et al., 2012) and prosocial behavior (Scrimgeour et al., 2013). Increased positive coparenting has also been moderately linked to increased academic achievement in school (Dopkins Stright and Neitzel, 2003; Cabrera et al., 2012), faster language development, and increased social functioning (Cheng et al., 2009). Negative coparenting has been linked with behavior problems (LeRoy et al., 2013), reduced communication and social skills (Nandy et al., 2021), poor child adjustment and later psychopathology (Umemura et al., 2015).

Coparenting has also been linked with parental mental health, with findings indicating that parental depression negatively impacts the coparenting relationship (Price-Robertson et al., 2017; Tissot et al., 2017; Williams, 2018; Turney and Hardie, 2021). Other research suggests that coparenting conflict increases depressive symptoms among mothers (Cabrera et al., 2012), which is in line with studies demonstrating a link between relationship conflict and parental anxiety and depression (Yap et al., 2014). These findings are also consistent with Feinberg and colleagues, who found that interventions targeting the coparenting relationship can reduce symptoms of depression and anxiety in mothers (Feinberg and Kan, 2008; Feinberg et al., 2016).

Recent research has hypothesized that coparenting may act as a mechanism through which anxiety is transmitted from parents to children, with study findings demonstrating correlations between parental anxiety and undermining coparenting, as well as between undermining coparenting and fearful temperaments in children (Metz et al., 2018). A similar finding has also been shown for maternal depression with one study finding that coparenting support mediated the relationship between maternal depression and child outcomes, with increased symptoms of depression linked to poorer coparental support, which then predicted an increase in behavior problems among children (Tissot et al., 2016).

Jessee et al. (2018) theorized that reflective functioning may be a protective factor during the transition to parenthood. Given this transition is often characterized by conflict and distress for new parents, a greater capacity to understand the emotional experiences underlying the behavior of themselves and their partner protects the couple relationship and the emerging coparenting relationship. Since the relational patterns that emerge during this period often endure throughout the remainder of the coparenting relationship, it is crucial to understand the factors that may underpin both successful and at risk coparenting relationships (Jessee et al., 2018). Several studies have found links between reflective functioning and coparenting or couple interactions. One such study followed a high-risk sample of pregnant women, finding that reflective functioning was associated with greater couple cohesion (Borelli et al., 2021). Similarly, other studies have also found a relationship between better parental reflective functioning and more positive coparenting relationships (Jessee, 2012; Marcu et al., 2016; Shai et al., 2017; Holtzinger, 2021).

While the examination of reflective functioning and coparenting together is growing, very few studies have gone a step further and examined how child outcomes fit within this picture. In their study, Jessee et al. (2018) recruited 103 couples who were followed longitudinally from pre-birth to 13 months post-birth. Findings suggested that maternal, but not paternal, reflective functioning predicted both supportive and undermining coparenting (Jessee et al., 2018). They also found that higher interparental conflict was associated with greater levels of anger and lower levels of enthusiasm and compliance in children. Reflective functioning was not found to be associated with any child outcome variable (Jessee et al., 2018). The authors hypothesized that this may have been due to the low stress nature of the 15-min family play task used, which may not have been sufficient for behaviors typically associated with poor reflective functioning to emerge (Jessee et al., 2018).

León and Olhaberry (2020) went a step further in their study, carrying out an exploratory mediation analysis which found that the quality of triadic interactions (the interaction between both parents and their infant, which includes coparenting) mediated the relationship between maternal but not paternal reflective functioning and child social emotional outcomes (León and Olhaberry, 2020). Fifty Chilean families whose 12 to 38 month old children had been referred for social–emotional difficulties participated in this study (León and Olhaberry, 2020). In addition to the novel mediation analysis, they also found that more positive triadic interactions were associated with higher levels of both maternal and paternal reflective functioning as well as fewer social emotional difficulties in children. The relationships between maternal and paternal reflective functioning and social emotional difficulties were not significant because this relationship was fully explained by triadic interactions. This study also found that when both mothers’ and fathers’ reflective functioning were included as predictors of triadic interactions, only mothers’ reflective functioning remained a significant predictor (León and Olhaberry, 2020).

It is of note that neither Jessee et al. (2018) nor León and Olhaberry (2020) included parental mental health as a variable within their studies. Given the established link between parental mental health difficulties and adverse child outcomes (McCall-Hosenfeld et al., 2016), it can be argued that parental mental health may be a significant piece of the puzzle linking parental reflective functioning, coparenting and child outcomes.

To our knowledge, Dollberg et al. (2021) were the first to include parental mental health, proposing a mediation-moderation hypothesis whereby coparenting would mediate the relationship between parental anxiety and child outcomes with parental reflective functioning acting as a moderator variable (Dollberg et al., 2021). They recruited 78 couples with children aged between 3 and 5, and found that coparenting did mediate the relationship between parent anxiety and child outcomes, however no support was found for reflective functioning as a moderator within this relationship (Dollberg et al., 2021). Findings suggested that reflective functioning did moderate the relationship between parental anxiety and child outcomes when coparenting was not included in the model (Dollberg et al., 2021). Reflective functioning was not found to be significantly correlated with any study variables with the exception of father’s reflective functioning which was significantly associated with father’s anxiety levels (Dollberg et al., 2021). The authors suggest that the low sample size may have contributed to the insignificant mediation-moderation hypothesis (Dollberg et al., 2021), therefore it may be worth examining whether this relationship exists in a larger sample of parents.

### The current study: Aims and hypotheses

The overall aim of the present study was to cross-sectionally investigate the variables involved in predicting child outcomes in early childhood, in particular, parental mental health, parental reflective functioning and coparenting and to examine how these variables are related to one another among parents. This is important to consider given the scarcity of research examining these variables together, particularly within a large sample of parents who have children in the period of early childhood. Given that the coparenting relationship emerges in early infancy, it is particularly worth examining how these variables interact in the first 4 years of the child’s life.

Informed by prior studies, we hypothesized that:

1. Poorer infant social emotional development will be predicted by higher levels of parental depression and anxiety, less positive and more negative coparenting and poorer general reflective functioning and parental reflective functioning.
2. Poorer parental reflective functioning will be predicted by poorer general reflective functioning, less positive and more negative coparenting and increased symptoms of depression and anxiety.
3. More negative and less positive coparenting will be predicted by poorer general reflective functioning and increased symptoms of depression and anxiety.
4. Increased symptoms of depression and anxiety will be predicted by poorer general reflective functioning.

## Materials and methods

### Methods

#### Design

The present study implemented a cross-sectional, correlational research design to examine associations between parental mental health, parental reflective functioning, coparenting, and child social emotional development.

#### Participants

Participants were 350 parents (175 women, 175 men) with children aged 0 to 3 years 11 months who were recruited via Prolific, an online recruiting platform. Inclusion criteria were met if the participant had a child in the correct age range and was in a relationship with and living with the other parent of their child. Participants were paid £3.75 GBP (roughly $7.15 AUD) through the Prolific website after completion of the questionnaire.

Participants’ ages ranged from 19 to 61 years (*M* = 33.63, *SD* = 5.31) and children were aged between 0 and 46 months (*M* = 21.29, *SD* = 12.77). 72.9% of the sample were married, 16.3% were engaged, 8.3% were in a defacto relationship and the remaining participants described their relationship status as other. This sample consisted predominantly of participants who identified as Caucasian (including British, European, American, Australian, or New Zealander; 82.5%). Other ethnicities included Asian or South East Asian (5.7%), Black (including African, African American, African British and Black Carribean) 4.3%, Hispanic 2.6%, South Asian (including Pakistani, Indian and Bangladeshi; 3.1%), Arabic or Islam 0.86%, mixed ethnicity 0.86%, while the final 0.29% of participants identified as Wichita or Native American.

In order to detect a medium size effect using a mediation analysis, research suggests a sample size of at least 300 participants is needed (Fritz and MacKinnon, 2007). Therefore, the present sample of 350 participants was deemed sufficient to detect at least medium-sized effects.

### Materials

The Depression Anxiety Stress Scale (DASS; Lovibond and Lovibond, 1995) is a 21-item self-report questionnaire measuring symptoms of depression, anxiety and stress over the past 7 days, across three 7-item subscales. Items are measured on a four-point Likert scale from 0 (“Did not apply to me at all”) to 3 (“Applied to me very much, or most of the time”) and are summed, with higher scores indicative of more severe symptoms. The DASS 21 is a widely used, well-validated scale that has demonstrated good internal reliability across its three subscales (Cronbach’s α = 0.81–0.88) as well as good convergent validity (*r* = 0.5–0.8) as shown by correlations between the DASS and other validated measures of depression and anxiety (Osman et al., 2012).

The 4-item Couples Satisfaction Index (CSI; Funk and Rogge, 2007) is a measure of relationship satisfaction, developed using item response theory. Responses are recorded on a 6- or 7-point Likert scale. Ratings are summed, with higher scores indicative of greater relationship satisfaction. This scale has shown good reliability (Cronbach’s α = 0.98) and convergent validity (*r* = 0.85–0.99) as shown by correlations between the CSI and other validated measures of relationship satisfaction and has been found sensitive to changes in relationship satisfaction (Funk and Rogge, 2007).

The Coparenting Relationship Scale (CRS; Feinberg et al., 2012) is 35-item self-report questionnaire that measures coparenting across 7 dimensions: agreement, endorsement, closeness, support and cooperation, division of labor, competition, undermining and the extent of child exposure to parental conflict. Items include “I believe my partner is a good parent” and “My partner undermines my parenting” and are rated on a 7-point scale from 0 (“Not true of us”) to 6 (“Very true of us”). Of the seven subscales, 5 focus on positive aspects of coparenting, while 2 (competition and undermining) focus on the more negative parts of the construct. Therefore, in our study, we created two subscales, positive coparenting and negative coparenting. Items were summed, and higher scores on the positive coparenting subscale indicate a more positive coparenting relationship, while higher scores on the negative coparenting subscale indicate greater levels of competition, undermining and parental conflict. This scale has shown good reliability (Cronbach’s α = 0.91–0.94) and construct validity (*r* = 0.60–0.74) as shown by correlations between the CRS and other related constructs (Feinberg et al., 2012).

The 8-item Reflective Functioning Questionnaire (RFQ; Fonagy et al., 2016) is a measure of mentalizing that is made up of two scales (certainty about mental states [RFQ_C] and uncertainty about mental states [RFQ_U]). This measure is scored on a 7-point Likert scale from 1 (“do not agree at all”) to 7 (“agree completely”). Items include “People’s thoughts are a mystery to me” and “I always know what I feel.” Adequate reliability has been demonstrated (Cronbach’s α ≥ 0.7), along with good construct validity shown through positive correlations between RFQ_U and the Toronto Alexithymia Scale (*r* = 0.66), and positive correlations between RFQ_C and the Kentucky Inventory of Mindfulness (*r* = 0.39; Cucchi et al., 2018).

The Parental Reflective Functioning Questionnaire (PRFQ-18; Luyten et al., 2017) is an 18-item self-report questionnaire that measures parental reflective functioning across three subscales. Subscales include: Pre-Mentalizing (e.g., “My child cries around strangers to embarrass me”), Certainty about Mental States (e.g., “I can always predict what my child will do”), Interest and Curiosity (e.g., I wonder a lot about what my child is thinking and feeling”). Items are measured on a 7-point Likert scale from 1 (“Strongly disagree”) to 7 (“Strongly agree”). This questionnaire has been related to attachment, sensitivity and parenting stress and has shown good reliability (Cronbach’s α = 0.79–0.85). Construct validity (*r* = 0.49) has been demonstrated through correlations between the Pre-Mentalizing subscale on the PRFQ-18 and both attachment anxiety and attachment avoidance measured with the Experience of Close Relationships-Revised, as well as correlations with other related constructs (Luyten et al., 2017).

The Ages and Stages Questionnaire: Social–Emotional (ASQ: SE 6; Squires et al., 2001) is a measure of social–emotional development in infants aged 3 to 65 months. There are specific forms for eight different age ranges. The number of items vary for each age range. This questionnaire includes 7 subscales: self-regulation, compliance, communication, autonomy affect, interaction with people, and adaptive functioning, with items measured on a 3-point Likert scale from (0 = “Most of the time,” 5 = “Sometime,” 10 = “Rarely or Never”). Mothers are also able to indicate whether the listed behavior is of concern. Five points are added to the total score if this option is ticked. Higher scores are indicative of more social–emotional problems on each respective dimension. Because of the varying number of items for each age group, total scores were averaged by dividing by total number of items on the form to enable comparison between age groups. These scales have been widely used in this area of research, and have demonstrated sufficient internal reliability and concurrent validity (Squires et al., 2001).

### Procedure

Ethics approval for the present study was granted by the Curtin University Human Research Ethics Committee (CUHREC). Following recruitment through Prolific, participants were redirected to a Qualtrics survey containing the study’s explanatory statement and all study measures. Participants then provided consent within Qualtrics before completing the online survey which took on average 30 min to complete.

Measures were preceded by several demographic questions (i.e., age, education level, and ethnicity and the final page of the survey provided a study debrief including links to support services). Participants were credited for their time upon valid completion of the survey.

### Data analysis plan

Analyses were run using both SPSS (v.28) and R statistical software (R Core Team, 2020). Our mediation model was run using the *Lavaan* package (Rosseel, 2012). We specified a sequential mediation model to assess the association between reflective functioning and child social emotional development. Using a sequential model in this way allows the relationship between mediators to be measured as well as allowing mediators to be predicted both by reflective functioning and by preceding mediator variables. Negative emotional symptoms (DASS scores) were included as the first mediator, parental reflective functioning (PRFQ scores) included as the second mediator, and finally coparenting (CRS scores) was included as the third mediator. Bias-corrected bootstrapped confidence intervals [10,000 iterations; as recommended by Hayes, (2017)] were used to test the indirect effect of reflective functioning on child social emotional development via each of these mediators.

## Results

### Correlations and descriptive analyses

To address issues of non-linearity, square root transformations were conducted for the DASS Anxiety subscale, the CSI, the RFQ Uncertainty subscale, the PRFQ Pre-mentalizing subscale, and the ASQ prior to model testing. The bivariate correlations and descriptive statistics are provided in Table 2. All of the variables with the exception of positive coparenting were significantly correlated with child social emotional development. Additionally, we observed a number of significant correlations between the predictor variables (see Table 2).

### Predicting child social emotional development

The variables included in this sequential mediation model accounted for a statistically significant 18.7% of the variance in child social emotional development, equating to a small-sized effect. The total effect of reflective functioning (Uncertainty subscale only) on child social emotional development was statistically significant (*b* = 0.171, *p* = 0.046, 95% CI: 0.002, 0.338).

#### Predictors of child development

Despite statistically significant bivariate associations with children’s social–emotional development (Table 1), both subscales of the RFQ as well as parental symptoms of depression, anxiety and stress were not significant predictors of child social emotional development in the final model that included the complete set of predictor variables.

**Table 1:** Descriptive statistics and correlations between measurement variables (*N* = 350).

	Correlations											Descriptives		
Variables	1	2	3	4	5	6	7	8	9	10	*11*	*M*	*SD*	*α*
1. Reflective functioning uncertainty	-	**-**	**-**	**-**	**-**	-	-	-	-	-	-	0.637	0.447	0.806
2. Reflective functioning certainty	**−0.737** ^**^	-	**-**	**-**	**-**	-	-	-	-	-	-	0.905	0.826	0.850
3. DASS depression	0.557^**^	−0.408^**^	-	**-**	**-**	-	-	-	-	-	-	4.418	3.989	0.891
4. DASS Anxiety	**0.498** ^**^	**0.424** ^**^	**0.611** ^**^	-	**-**	-	-	-	-	-	-	1.363	1.026	0.839
5. DASS stress	**0.606** ^**^	**−0.504** ^**^	**0.716** ^**^	**0.700** ^**^	-	-	-	-	-	-	-	6.650	4.212	0.877
6. Parental reflective functioning pre-mentalizing	0.238**	−0.370**	0.240**	0.249**	0.198**	-	-	-	-	-	-	1.385	0.302	0.753
7. Parental reflective functioning certainty	**−0.257** ^**^	−0.210*	**−0.194** ^**^	−0.122*	−0.208**	**−0.176** ^**^	-	-	-	-	-	4.002	1.057	0.783
8. Parental reflective functioning interest and curiosity	−0.057	0.087	−0.010	0.052	0.036	−0.403**	0.189**	-	-	-	-	5.645	0.738	0.674
9. Positive coparenting	−0.243**	0.285**	−0.317**	−0.254**	−0.241**	−0.328**	0.085	0.225**	-	-	-	108.611	24.425	0.939
10. Negative coparenting	0.311**	−0.321**	0.373**	0.353**	0.328**	0.426**	−0.043	−0.192**	−0.619**	-	-	11.304	10.331	0.890
11. Relationship satisfaction	0.190*	−0.208**	0.303**	0.188**	0.236**	0.210**	−0.107*	−0.118*	−0.726**	0.476**	-	2.609	0.879	0.950
12. Child social emotional development	0.246**	−0.237**	0.220**	0.236**	0.191**	0.340**	−0.209**	−0.165**	−0.097	0.223**	0.026	1.273	0.493	0.336–0.912

Bivariate correlations are presented on the lower quadrant. ^**^p < 0.001, ^*^p < 0.05. Bold values indicate statistical significance.

Negative coparenting was also not a significant predictor. However, positive coparenting remained a significant predictor in the final model, albeit with a small effect size (*b* = 0.003, *p* = 0.043, 95% CI: 0.000, 0.005).

The Pre-mentalizing (*b* = 0.373, *p* = 0.002, 95% CI: 0.136, 0.601) and Certainty (*b* = −0.057, *p* = 0.037, 95% CI: −0.109, −0.003) subscales of the PRFQ, but not Interest and Curiosity, were also found to be significant predictors of child social emotional development. Of the variables examined in the present study, the pre-mentalizing subscale of the PRFQ was the most significant predictor of child social emotional development (see Table 2).

**Table 2:** Predictors of child social emotional development, with 95% Bias corrected confidence intervals reported in parenthesis.

Variables	*B* (95% CI)	*SE B*	Std. All	*p*
C1—Reflective functioning uncertainty	0.131 (−0.053, 0.317)	0.094	0.119	0.163
C2—Reflective functioning certainty	0.000 (−0.089, 0.095)	0.047	0.000	0.997
B1—Positive coparenting	0.003 (0.000, 0.005)	0.001	0.131	**0.043**
B2—Negative coparenting	0.006 (−0.001, 0.012)	0.003	0.115	0.086
B3—DASS anxiety	0.058 (−0.015, 0.134)	0.038	0.119	0.130
B4—DASS depression	0.007 (−0.011, 0.025)	0.009	0.055	0.464
B5—DASS stress	−0.009 (−0.029, 0.010)	0.010	−0.079	0.338
B6—Parental reflective functioning pre-mentalizing	0.373 (0.136, 0.601)	0.119	0.228	**0.002**
B7—Parental reflective functioning certainty	−0.057 (−0.109, −0.003)	0.027	−0.121	**0.037**
B8—Parental reflective functioning interest and curiosity	−0.044 (−0.124, 0.035)	0.040	−0.065	0.276
Total effect of RFQ uncertainty	0.171 (0.002, 0.338)	0.085	0.154	**0.046**
Total effect of RFQ certainty	−0.06 (−0.143, 0.029)	0.044	−0.101	0.172

R^2^ = 0.187. Confidence intervals and standard errors based on 10,000 Bootstrap samples (*N* = 350). Bold values indicate statistical significance.

### Predictors of parental reflective functioning

The RFQ Uncertainty subscale was found to significantly predict all three PRFQ subscales: Pre-mentalizing (*b* = −0.103, *p* = 0.050, 95% CI: −0.206, 0.000), Certainty (*b* = −0.454, *p* = 0.047, 95% CI: −0.890, −0.001) and Interest and Curiosity (*b* = 0.452, *p* = 0.001, 95% CI: 0.183, 0.720). Whereas the RFQ Certainty subscale was found to only significantly predict two PRFQ subscales: Pre-mentalizing (*b* = −0.137, *p* = 0.000, 95% CI: −0.183, −0.090), Interest and Curiosity (*b* = 0.225, *p* = 0.002, 95% CI: 0.077, 0.368). Negative coparenting was also found to predict the Pre-mentalizing subscale of the PRFQ (*b* = 0.009, *p* = 0.000, 95% CI: 0.005, 0.013) while Positive coparenting was found to predict the Interest and Curiosity subscale of the PRFQ (*b* = 0.005, *p* = 0.038, 95% CI: 0.000, 0.009). Parental symptoms of depression, anxiety and stress were not found to predict parental reflective functioning in the present study (see Table 3).

**Table 3:** Predictors of parental reflective functioning, with 95% bias corrected confidence intervals reported in parenthesis.

	Variables	*b*	*SE B*	Std. All	*p*
**Pre-mentalizing**	A12—Reflective functioning uncertainty	−0.103 (−0.206, 0.000)	0.052	−0.151	**0.050**
	A13—Reflective functioning certainty	−0.137 (−0.183, −0.090)	0.024	−0.372	**0.000**
	A14—Positive coparenting	−0.001 (−0.002, 0.001)	0.001	−0.046	0.461
	A35—Negative coparenting	0.009 (0.005, 0.013)	0.002	0.292	**0.000**
	A15—DASS anxiety	0.030 (−0.009, 0.068)	0.020	0.102	0.122
	A16—DASS depression	0.005 (−0.005, 0.016)	0.005	0.070	0.317
	A17—DASS stress	−0.009 (−0.021, 0.002)	0.006	−0.130	0.108
**Certainty**	A18—Reflective functioning uncertainty	−0.454 (−0.890, −0.001)	0.228	−0.191	**0.047**
	A19—Reflective functioning certainty	0.039 (−0.188, 0.272)	0.116	0.030	0.737
	A20—Positive coparenting	0.003 (−0.003, 0.009)	0.003	0.064	0.338
	A36—Negative coparenting	0.008 (−0.007, 0.023)	0.008	0.080	0.273
	A21—DASS anxiety	0.098 (−0.051, 0.243)	0.075	0.095	0.189
	A22—DASS depression	−0.018 (−0.063, 0.029)	0.024	−0.068	0.444
	A23—DASS stress	−0.027 (−0.073, 0.015)	0.022	−0.109	0.216
**Interest and curiosity**	A24—Reflective functioning uncertainty	0.452 (0.183, 0.720)	0.137	0.274	**0.001**
	A25—Reflective functioning certainty	0.225 (0.077, 0.368)	0.074	0.252	**0.002**
	A26—Positive coparenting	0.005 (0.000, 0.009)	0.002	0.159	**0.038**
	A37—Negative coparenting	−0.008 (−0.020, 0.003)	0.006	−0.113	0.157
	A27—DASS anxiety	0.074 (−0.030, 0.177)	0.053	0.103	0.159
	A28—DASS depression	−0.013 (−0.044, 0.019)	0.016	−0.072	0.402
	A29—DASS stress	0.008 (−0.023, 0.038)	0.016	0.048	0.591

Pre-mentalizing R^2^ = 0.268, Certainty R^2^ = 0.095, Interest and curiosity R^2^ = 0.097. Confidence intervals and standard errors based on 10,000 bootstrap samples (*N* = 350). Bold values indicate statistical significance.

#### Predictors of coparenting

The certainty subscale (but not the uncertainty subscale) of the RFQ was found to predict both positive (*b* = 7.647, *p* = 0.001, 95% CI: 2.820, 12.232) and negative (*b* = −2.667, *p* = 0.004, 95% CI: −4.422, −0.803) coparenting. Of the DASS subscales, only symptoms of depression were found to predict both positive (*b* = −1.698, *p* = 0.001, 95% CI: −2.672, −0.686) and negative (*b* = 0.664, *p* = 0.002, 95% CI: 0.243, 1.080) coparenting, while symptoms of anxiety were found to predict negative coparenting only (*b* = 1.900, *p* = 0.010, 95% CI: 0.468, 3.364; see Table 4).

**Table 4:** Predictors of coparenting, with 95% bias corrected confidence intervals reported in parenthesis.

	Variables	*b* (95% CI)	*SE B*	Std. All	*p*
**Positive coparenting**	A1—Reflective functioning uncertainty	5.207 (−4.389, 14.274)	4.732	0.094	0.271
	A2—Reflective functioning certainty	7.647 (2.820, 12.232)	2.402	0.256	**0.001**
	A5—DASS anxiety	−2.501 (−6.058, 0.901)	1.787	−0.104	0.162
	A8—DASS depression	−1.698 (−2.672, −0.686)	0.503	−0.274	**0.001**
	A11—DASS stress	0.633 (−0.436, 1.699)	0.544	0.108	0.245
**Negative coparenting**	A30—Reflective functioning uncertainty	−1.476 (−4.940, 2.186)	1.807	−0.065	0.414
	A31—Reflective functioning certainty	−2.667 (−4.422, −0.803)	0.917	−0.216	**0.004**
	A32—DASS anxiety	1.900 (0.468, 3.364)	0.737	0.192	**0.010**
	A33—DASS depression	0.664 (0.243, 1.080)	0.211	0.260	**0.002**
	A34—DASS stress	−0.166 (−0.562, 0.242)	0.207	−0.069	0.422

Positive coparenting R^2^ = 0.151, Negative coparenting R^2^ = 0.188. Confidence intervals and standard errors based on 10,000 bootstrap samples (*N* = 350). Bold values indicate statistical significance.

#### Predictors of parental mental health

The uncertainty subscale of the reflective functioning questionnaire was found to predict DASS symptoms of anxiety (*b* = 0.946, *p* = 0.000, 95% CI: 0.615, 1.297), depression (*b* = 5.051, *p* = 0.000, 95% CI: 3.658, 6.402) and stress (*b* = 4.815, *p* = 0.000, 95% CI: 3.537, 6.043), while the certainty subscale predicted symptoms of stress only (*b* = −0.674, *p* = 0.039, 95% CI: −1.291, −0.011; see Table 5).

**Table 5:** Predictors of depression, anxiety and stress, with 95% bias corrected confidence intervals reported in parenthesis.

	Variables	*b*	*SE B*	Std. All	*p*
**Anxiety**	A3—Reflective functioning uncertainty	0.946 (0.615, 1.297)	0.174	0.412	**0.000**
	A4—Reflective functioning certainty	−0.151 (−0.327, 0.034)	0.093	−0.122	0.103
**Depression**	A6—Reflective functioning uncertainty	5.051 (3.658, 6.402)	0.695	0.566	**0.000**
	A7—Reflective functioning certainty	0.033 (−0.608, 0.670)	0.323	0.007	0.919
**Stress**	A9—Reflective functioning uncertainty	4.815 (3.537, 6.043)	0.632	0.511	**0.000**
	A10—Reflective functioning certainty	−0.674 (−1.291, −0.011)	0.327	−0.132	**0.039**

Anxiety R^2^ = 0.258, Depression R^2^ = 0.315, Stress R^2^ = 0.378. Confidence Intervals and standard errors based on 10,000 bootstrap samples (*N* = 350). Bold values indicate statistical significance.

### Exploratory indirect effect analyses

We performed a number of analyses to determine whether any indirect effects were present. In particular we explored whether there was an indirect effect of general reflective functioning on child social emotional development via parental reflective functioning. In the present study, there was an indirect effect of the certainty subscale of the RFQ on child social emotional development via the Pre-mentalizing subscale of the PRFQ (*b* = −0.051, *p* = 0.009, 95% CI:-0.093, −0.017). The remaining mediation analyses explored were not significant (see Table 6).

**Table 6:** Indirect effects of general reflective functioning on child social emotional development via parental reflective functioning, with 95% bias corrected confidence intervals reported in parenthesis.

Variables	*b*	*SE B*	Std. All	*p*
Indirect pathway from RFQ (uncertainty) to ASQ via PRFQ (pre-mentalizing).	−0.038 (−0.098, 0.000)	0.025	−0.035	0.130
Indirect pathway from RFQ (certainty) to ASQ via PRFQ (pre-mentalizing).	−0.051 (−0.093, −0.017)	0.019	−0.085	**0.009**
Indirect pathway from RFQ (uncertainty) to ASQ via PRFQ (certainty).	0.026 (−0.003, 0.068)	0.019	0.023	0.170
Indirect pathway from RFQ (certainty) to ASQ via PRFQ (certainty).	−0.002 (−0.019, 0.012)	0.007	−0.004	0.767
Indirect pathway from RFQ (uncertainty) to ASQ via PRFQ (interest and curiosity).	−0.02 (−0.064, 0.016)	0.020	−0.018	0.325
Indirect pathway from RFQ (certainty) to ASQ via PRFQ (interest and curiosity).	−0.01 (−0.029, 0.009)	0.009	−0.016	0.299

Confidence intervals and standard errors based on 10,000 bootstrap samples (*N* = 350). Bold values indicate statistical significance.

We also explored whether there was an indirect effect of general reflective functioning on child social emotional development via coparenting. This was not found to be the case, however there was an indirect effect of negative coparenting on child social emotional development via the PRFQ Pre-mentalizing subscale (*b* = 0.003, *p* = 0.011, 95% CI: 0.001, 0.006). The remaining mediation analyses explored were not significant (see Table 7).

**Table 7:** Indirect effects of general reflective functioning on child social emotional development via coparenting, with 95% bias corrected confidence intervals reported in parenthesis.

Variables	*b*	*SE B*	Std. All	*p*
Indirect pathway from RFQ (uncertainty) to ASQ via positive coparenting.	0.014 (−0.011, 0.050)	0.016	0.012	0.382
Indirect pathway from RFQ (certainty) to ASQ via positive coparenting.	0.02 (0.000, 0.048)	0.012	0.033	0.107
Indirect pathway from RFQ (uncertainty) to ASQ via negative coparenting.	−0.008 (−0.041, 0.013)	0.013	−0.007	0.536
Indirect pathway from RFQ (certainty) to ASQ via negative coparenting.	−0.015 (−0.039, 0.001)	0.011	−0.025	0.160
Indirect pathway from negative coparenting to ASQ via PRFQ (pre-mentalizing).	0.003 (0.001, 0.006)	0.001	0.067	**0.011**

Confidence intervals and standard errors based on 10,000 bootstrap samples (*N* = 350). Bold values indicate statistical significance.

Finally, we explored whether there would be an indirect effect of symptoms of depression and anxiety on child social emotional development via coparenting. As seen in Table 8, we did not find any support for this hypothesis, with all *p* values found to be above the 0.05 cut-off for statistical significance.

**Table 8:** Indirect effects of DASS subscales on child social emotional development via coparenting, with 95% bias corrected confidence intervals reported in parenthesis.

Variables	*b*	*SE B*	Std. All	*p*
Indirect pathway from DASS depression to ASQ via positive coparenting	−0.004 (−0.011, 0.000)	0.003	−0.036	0.109
Indirect pathway from DASS depression to ASQ via negative coparenting	0.004 (0.000, 0.010)	0.003	0.030	0.153
Indirect pathway from DASS anxiety to ASQ via positive coparenting	−0.007 (−0.021, 0.002)	0.006	−0.014	0.288
Indirect pathway from DASS anxiety to ASQ via negative coparenting	0.011 (−0.001, 0.028)	0.008	0.022	0.162
Indirect pathway from DASS stress to ASQ via positive coparenting	0.002 (−0.001, 0.006)	0.002	0.014	0.382
Indirect pathway from DASS stress to ASQ via negative coparenting	−0.001 (−0.004, 0.002)	0.001	−0.008	0.523

Confidence intervals and standard errors based on 10,000 bootstrap samples (*N* = 350).

## Discussion

The overall aim of the present study was to cross-sectionally investigate the variables involved in predicting child outcomes in early childhood. The specific aims of the present study were to investigate relationships between parental mental health, parental reflective functioning, coparenting and child social emotional development in both mothers and fathers during early childhood. Surprisingly, the present study found that both general reflective functioning and parental symptoms of depression, anxiety and stress were not significant predictors of child social emotional (SE) development. However, in line with our expectations both coparenting (positive) and parental reflective functioning (in particular Pre-mentalizing and Certainty) were found to significantly predict child SE development.

As anticipated, general reflective functioning (uncertainty subscale only) predicted symptoms of depression and anxiety, while parental depression and anxiety were both predictors of coparenting (anxiety predicted negative coparenting only). General reflective functioning (certainty only) was also found to predict coparenting. Coparenting in turn was found to predict the parental reflective functioning (positive coparenting predicted PRFQ Pre-mentalizing, while negative coparenting predicted PRFQ Interest and Curiosity). Interestingly, parental reflective functioning was not predicted by parental depression and anxiety in the present study, but was predicted by general reflective functioning.

Given the pattern of findings that were identified, in conjunction with some preliminary suggestions in further research, some exploratory tests of indirect associations were carried out. Of note, we found an indirect effect of general reflective functioning (certainty) on child SE development via parental reflective functioning (Pre-mentalizing). We also found an indirect effect of negative coparenting on child SE development via parental reflective functioning (Pre-mentalizing). We did not however find any indirect effects between depression and anxiety, coparenting and child SE development. The current results support a growing body of research highlighting the important role reflective functioning plays in child development and wellbeing as well as parental mental health and the interparental relationship.

The significant relationship found in our study between parental reflective functioning and child SE development was anticipated given prior research demonstrating links between higher maternal and paternal reflective functioning and better social emotional adjustment in children (Gordo et al., 2020; Salo et al., 2021). In particular, we found that higher scores on the pre-mentalizing subscale of the PRFQ were associated with poorer SE development. This makes sense given that increased levels of pre-mentalizing modes in caregivers are indicative of severe mentalizing difficulties (Luyten et al., 2017). This is often displayed as high levels of certainty about a child’s mental state which may cause parents to attribute false malevolent intentions to a child’s difficult behaviors (e.g., “my child cries around strangers to embarrass me”; Luyten et al., 2017). These parents may also have difficulty understanding their child’s internal world (Luyten et al., 2017).

Interestingly, in the present study greater certainty about mental states (as shown by the Certainty subscale of the PRFQ) was linked with fewer social emotional symptoms. It is important to note that very high levels of certainty about mental states may suggest intrusive mentalizing (also known as hypermentalizing), whereby the parent does not recognize that it is not possible to fully comprehend the mental states of others (e.g., mental states are opaque) while very low levels of certainty may indicate hypomentalizing (a very poor understanding of one’s child’s mental states; Luyten et al., 2017). Therefore, better parental reflective functioning would be shown by scores in the mid-range on this subscale of the PRFQ. Given that parental reflective functioning is thought to be essential to children developing both emotion regulation skills and a secure parent-infant attachment relationship (Ordway et al., 2015) our results are overall in line with expectations based on what has been shown in the literature.

The significant relationship found between higher levels of positive coparenting and better child SE development (*b* = 0.003, *p* = 0.043) was also anticipated given the large body of research linking coparenting with later child adjustment (Teubert and Pinquart, 2010; Umemura et al., 2015). This is thought to be because better coparenting is a key predictor of overall family functioning, and may lead to reduced interparental conflict and stress and more consistent and sensitive parenting (Feinberg et al., 2010; Dollberg et al., 2021).

Based on prior research, we also hypothesized that reflective functioning would be a key variable involved in predicting coparenting, and this was found to be the case. In particular, higher levels of certainty about mental states were linked with more positive coparenting and less negative coparenting. This is unsurprising given prior research which has found associations between higher reflective functioning and better coparenting quality (Jessee, 2012; Marcu et al., 2016; Shai et al., 2017; Borelli et al., 2021; Holtzinger, 2021). It is thought that higher levels of reflective functioning should enable increased understanding of a spouse’s emotional experience and perspective, which in turn may assist couples to better manage conflict and repair ruptures in their relationship (Jessee et al., 2018).

We also reasoned that having a strong coparenting relationship may support the development of parental reflective functioning, and this was again supported in our results. We found that more positive coparenting predicted fewer mentalizing difficulties as shown through lower levels of pre-mentalizing modes, while more negative coparenting predicted less interest and curiosity about their infant’s internal world. It makes sense that this reciprocal relationship would exist between coparenting and reflective functioning, whereby strong reflective capacity enhances one’s ability to work well in a parenting team and that in turn supports more ability to be reflective about a child’s internal world.

**Figure 1:**
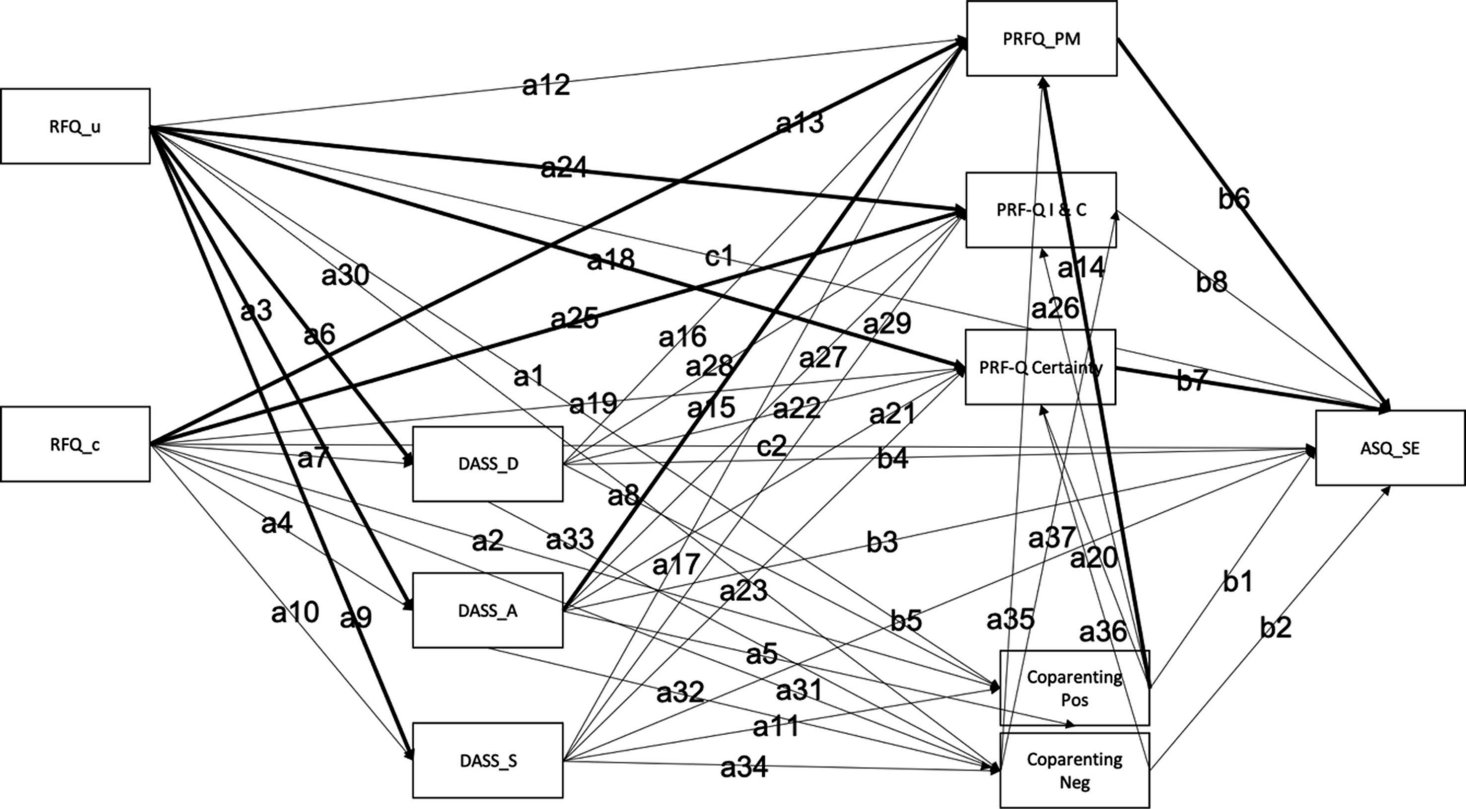
Relationship between parental depression and anxiety, reflective functioning, parental reflective functioning, coparenting and child social emotional symptoms. RFQ, reflective functioning questionnaire; DASS, depression, anxiety and stress scale; PRF-Q, parental reflective functioning questionnaire; ASQ-SE, ages and stages questionnaire—social emotional.

Surprisingly, in the present study, parental symptoms of depression, anxiety and stress were not significant predictors of child SE development. This was unexpected given the large body of research that has previously shown associations between parental depression and anxiety and child outcomes (McCall-Hosenfeld et al., 2016). We hypothesize that this may be because previous studies examining parental mental health as a predictor of child outcomes have not also considered other significant predictors such as reflective functioning and coparenting which both explain a higher percentage of the variance in child SE development. This would make sense, given the statistically significant bivariate associations observed between parental depression and anxiety and children’s SE development. As anticipated, our community sample had generally low levels of parental depression and anxiety. In fact, 75.4% of our sample were considered to have normal to mild symptoms of depression (60.4% of these fell in the normal range), while 82.6% of participants had normal to mild symptoms of anxiety (69.7% fell in the normal range). Parents in our sample also predominantly self-reported that their children had few SE difficulties. It is therefore possible that the low-risk nature of our sample reduced our capacity to pick up on the relationship between parental mental health and child SE development. It is therefore likely that these variables remain relevant, but may be less important as predictors in a general community sample when exampled alongside other important predictor variables (Figure 1).

In line with our expectations, we did find that poorer general reflective functioning (as demonstrated by higher levels of uncertainty about mental states) predicted greater symptoms of depression and anxiety. This is consistent with a body of research demonstrating general associations between poorer reflective functioning and higher levels of depression and anxiety (Fischer-Kern et al., 2013; Belvederi Murri et al., 2017; Bigelow et al., 2018; Wendelboe et al., 2021). This is thought to be because biases in reflective processes are thought to prevent an individual from regulating their intense emotional experiences or modulating the behavioral expression of these emotions (Bouchard et al., 2008; Luyten et al., 2013; Luyten and Fonagy, 2018). In turn, we also hypothesized that parental mental health may act as a predictor for parental reflective functioning, whereby it is easier to reflect on your child’s inner world when your own mental health is stronger. However, once all variables were entered into our final model, this relationship was no longer significant. It may be that this relationship does not show up with the self-report measures used in the present study, or it could be the case that other variables such as emotion regulation (Schultheis et al., 2019), and attachment history (Suchman et al., 2011) play a larger role in predicting parental reflective functioning.

Parental depression and anxiety were also found to predict coparenting such that higher levels of parental depression were associated with less positive and more negative coparenting, while higher levels of parental anxiety were associated with more negative coparenting. This is in line with a body of research suggesting that parental depression and anxiety negatively impact the coparenting relationship (Price-Robertson et al., 2017; Tissot et al., 2017; Metz et al., 2018; Williams, 2018; Turney and Hardie, 2021). This makes sense given that both executive functioning and reflective capacity are so impaired by poor mental health, and these factors make it harder to see a partner’s perspective and work well as a parenting team.

We also found that parental reflective functioning was predicted by general reflective functioning such that higher levels of RFQ uncertainty and lower levels of RFQ certainty predicted increased scores on the PRFQ pre-mentalizing modes. This makes sense given that high levels of pre-mentalizing modes are indicative of a lack of reflective capacity, in the same way that very high uncertainty and low certainty may indicate difficulties with mentalizing (Luyten et al., 2017). We also found that higher levels of RFQ uncertainty predicted lower scores on the PRFQ certainty subscale, which once again makes conceptual sense. Finally, increased RFQ uncertainty predicted less PRFQ interest and curiosity, while more RFQ certainty predicted greater PRFQ interest and curiosity. High levels of interest and curiosity are suggestive of greater reflective capacity, and as such this finding is in line with what we would expect to see. Given that most prior studies that have examined reflective functioning or parental reflective functioning have done so using observational or interview measures, few studies have examined how the RFQ and PRFQ are related among parents of young children. However, these results are all in the expected direction and make sense from a conceptual perspective.

In the present study we also carried out some exploratory mediation analyses, and found an indirect effect of general reflective functioning (certainty) on child SE development via parental reflective functioning (Pre-mentalizing). We found that greater certainty about mental states was associated with lower pre-mentalizing modes, which in turn was associated with better child SE development. General reflective functioning was not found to be a significant predictor of child SE development, however this is likely because the relationships between general reflective functioning and child SE development is fully explained by parental reflective functioning.

Given prior research suggesting that coparenting may act a mediator for the relationships between anxiety and depression and child outcomes (Tissot et al., 2016; Metz et al., 2018), we explored whether this would be the case in the present study. However, we did not find any evidence of an indirect effect of parental mental health on child SE development via coparenting. This may be because neither parental depression, anxiety or coparenting were strong predictors of child SE development once entered into our complete model, and therefore these relationships may have been overshadowed by stronger predictor variables. Or it could be the nature of the self-report measures included in the current study and the fact that on the whole our community sample had generally low levels of parental depression and anxiety as well as child SE difficulties, which may have reduced our ability to detect this relationship.

Unlike León and Olhaberry (2020) we also did not find an indirect effect of general reflective functioning on child SE development via coparenting, however given the exploratory nature of this part of our analysis we also considered some alternate pathways. In doing so, we found an indirect effect of negative coparenting on child SE development via parental reflective functioning (Pre-mentalizing). This effect is such that more negative coparenting predicted higher pre-mentalizing modes, which in turn was associated with worse child SE development. This makes sense given the likely reciprocal relationship between reflective functioning and coparenting, whereby the presence of a strong parenting team is likely to support stronger reflective capacity, especially in the context of parenting. We found that negative coparenting was not a significant predictor of child SE development, and once again, this is likely because the relationship between negative coparenting and child SE development is fully explained by parental reflective functioning, which overall has shown up in our study as the strongest predictor of child development.

### Strengths, limitations, and future directions

Our study is strengthened by our inclusion of both mothers and fathers, and an adequately-sized sample that allowed us to examine a range of key variables (parental mental health, coparenting, both general and parental reflective functioning) that are thought to predict child SE development. Nevertheless, our findings do need to be considered in light of several limitations. Firstly, the cross-sectional nature of this data prevents us from drawing causal inferences between study variables. The order in which we tested our variables was informed by prior literature and theoretical considerations, however these analyses alone are unable to make an inference of causality. For example, we argue that poor general reflective functioning may lead to increased risk of experiencing depression and anxiety, however there is also evidence suggesting that while experiencing depression and anxiety, an individual’s reflective processes are impeded (Luyten et al., 2013; Luyten and Fonagy, 2018). The same is true for the relationship between depression and anxiety and coparenting. We argue that poor mental health is likely to lead to a worse coparenting relationship, however there is also a body of research suggesting that coparental conflict may predict declining mental health (Cabrera et al., 2012). Given these considerations, we recognize that causal inferences cannot be drawn solely from this cross-sectional data. However, we hope that the findings presented in this paper will inform future more resource intensive longitudinal studies.

Another limitation within our study is our sole reliance on self-report measures for all study variables. In particular, coparenting, reflective functioning and child SE development are likely to be more accurately measured via observational tasks. This is because parents may lack the insight to answer accurately, or may attempt to portray a more favorable image of themselves and their coparental and parent–child relationships. Future research examining the relationship between these variables would benefit from including additional methods of data collection such as behavioral observation or interviews. Our data is also limited by the fact that while we included both fathers and mothers, we did not recruit couples and therefore we are limited in the inferences we can draw about how one parent’s reflective functioning may influence the other parent and in turn did not have an additional source of data on either the coparenting relationship or child SE development (i.e., the other parent may view the coparenting relationship or child’s level of development differently).

Our study also recruited participants from Western countries with a majority of participants identifying as Caucasian, thus some caution should be applied when attempting to generalize these findings into other cultural settings. Future research may wish to consider investigating how coparenting and reflective functioning relate to child SE development in different cultural contexts, given prior research establishing cultural differences in child care practices (Chen et al., 1998; Rosenthal and Roer-Strier, 2001).

Finally, the predictors examined in the present study explained only 18.7% of the variance in child SE development, which is a relatively small proportion of variance. This leaves 81.3% of the variance unexplained by the predictors considered in this study. This would suggest that numerous other variables are involved in predicting child outcomes, and future research may wish to consider additional factors that may be important to social emotional development in young children. In particular it may be important to consider variables such as the social support available, maternal and paternal attachment style, level of parental self-efficacy and stress as well as parental self-compassion.

### Implications

This study adds to a small but growing body of research investigating how both coparenting and reflective functioning interact to predict child outcomes. We are one of the first studies to demonstrate that reflective functioning is a key predictor of the coparenting relationship. We are also one of the first studies to consider how parental mental health fits into this picture. Parental mental health, and maternal depression in particular, has long been considered a key risk factor for the development of adverse child outcomes, and therefore targeting maternal depression has been a key focus of many public health initiatives during the perinatal period. Our results appear to suggest that parental reflective functioning is one of the most important predictors of child outcomes over and above parental mental health. Current interventions designed to improve parental reflective functioning, both group-based and dyadic, are still being refined and there is limited evidence for their effectiveness (Barlow et al., 2021; Lo and Wong, 2022). The findings of the current study support the continued development of these interventions as they indicate changes in parental reflective functioning may contribute to changes in child outcomes.

Our findings suggest that parental reflective functioning appears to play a large role in developing both a strong coparenting relationship and also supporting child social emotional development. Therefore, we hope these findings will inform future research and enable the continued development of early interventions for new parents that specifically target their reflective capacity. Targeting reflective functioning is likely to in turn reduce symptoms of poor mental health, improve coparenting and general family functioning and most importantly enable optimal social emotional development in infants and young children.

## Data availability statement

The raw data supporting the conclusions of this article will be made available by the authors, without undue reservation.

## Ethics statement

The studies involving human participants were reviewed and approved by Curtin Human Research Ethics Committee. The patients/participants provided their written informed consent to participate in this study.

## Author contributions

MD conceptualized and carried out the research project, including the selection of study variables, data collection, and data analysis and was the principal author of this publication. RR, EI, VM, and RK were the supervisors of the study, assisting in designing and conducting the research, and providing feedback on the publication. All authors contributed to the article and approved the submitted version.

## Conflict of interest

The authors declare that the research was conducted in the absence of any commercial or financial relationships that could be construed as a potential conflict of interest.

## Publisher’s note

All claims expressed in this article are solely those of the authors and do not necessarily represent those of their affiliated organizations, or those of the publisher, the editors and the reviewers. Any product that may be evaluated in this article, or claim that may be made by its manufacturer, is not guaranteed or endorsed by the publisher.
